# Draft Genome Sequence of *Kocuria palustris* PEL

**DOI:** 10.1128/genomeA.01261-13

**Published:** 2014-02-06

**Authors:** Gaurav Sharma, Indu Khatri, Srikrishna Subramanian

**Affiliations:** CSIR-Institute of Microbial Technology, Chandigarh, India

## Abstract

We report the 2.9-Mb draft genome sequence of an actinobacterium, *Kocuria palustris* PEL, of the family *Micrococcaceae*.

## GENOME ANNOUNCEMENT

*Kocuria palustris* is an aerobic Gram-positive spherical actinobacterium belonging to the family *Micrococcaceae* and suborder *Micrococcineae*. Bacteria of this family are found in diverse niches such as soil samples, clinical specimens, fermented food, and oral and skin flora. However, only a limited amount of genomic information is currently available for this family.

The genome of *K. palustris* PEL was sequenced using Illumina HiSeq 1000 technology. Sequencing resulted in 23,270,366 paired-end reads (insert size of 350 bp) of 101 bp in length. A total of 22,985,973 high-quality reads with approximately 800× coverage were assembled with CLC bio Genomics Workbench 5.5 (http://www.clcbio.com) (word size, 40; bubble size, 100) and generated 55 contigs (N_50_, 140,153 bp). Functional annotation was carried out by Rapid Annotations using Subsystems Technology (RAST) (1), tRNAs were predicted by tRNAscan-SE-1.23 (2), and rRNA genes were predicted by RNAmmer 1.2 (3). The genome contains 3 rRNA genes (5S-16S-23S) and 48 aminoacyl-tRNA synthetase genes. The draft genome of *K. palustris* PEL consists of 55 contigs of 2,872,112 bp, with an average G+C content of 70.4%.

The genome coding density is 88%, with an average gene length of 972 bp. A total of 2,561 coding regions were found in the genome, of which 1,814 (71%) were functionally annotated. The number of genes transcribed from the positive strand was 1,308, and 1,253 were transcribed from the negative strand. The annotated genome has 42 genes involved in virulence, disease, and defense, including 25 genes for resistance to antibiotics and toxic compounds. Sixty-nine genes are involved in the stress response of the bacterium, including 28 genes for oxidative stress and 20 genes functioning against osmotic stress.

The functional comparison of the genome sequences available on the RAST server revealed the closest neighbor of *K. palustris* PEL to be *Kocuria rhizophila* DC2201 (score, 516), followed by *Arthrobacter* sp. strain FB24 (score, 338), *Arthrobacter chlorophenolicus* A6 (score, 326), and *Renibacterium salmoninarum* ATCC 33209 (score, 319).

### Nucleotide sequence accession numbers.

This whole-genome shotgun project has been deposited at DDBJ/EMBL/GenBank under the accession no. ANHZ00000000. The version described in this paper is the second version, ANHZ02000000.
